# StRAP2.3, an ERF‐VII transcription factor, directly activates *StInvInh2* to enhance cold-induced sweetening resistance in potato

**DOI:** 10.1038/s41438-021-00522-1

**Published:** 2021-04-01

**Authors:** Weiling Shi, Yuhao Song, Tiantian Liu, Qiuqin Ma, Wang Yin, Yuchen Shen, Tengfei Liu, Chunyan Jiang, Kai Zhang, Dianqiu Lv, Botao Song, Jichun Wang, Xun Liu

**Affiliations:** 1 Chongqing Key Laboratory of Biology and Genetic Breeding for Tuber and Root Crops; College of Agronomy and Biotechnology, Southwest University, 400715 Chongqing, People’s Republic of China; 2 Key Laboratory of Horticultural Plant Biology (HZAU), Ministry of Education; Key Laboratory of Potato Biology and Biotechnology, Ministry of Agriculture and Rural Affairs, Huazhong Agricultural University, 430070 Wuhan, People’s Republic of China

**Keywords:** Molecular biology, Plant sciences

## Abstract

Potato invertase inhibitor (*StInvInh2*) positively regulates cold-induced sweetening (CIS) resistance by inhibiting the activity of vacuolar invertase. The distinct expression patterns of *StInvInh2* have been thoroughly characterized in different potato genotypes, but the related CIS ability has not been characterized. The understanding of the regulatory mechanisms that control *StInvInh2* transcription is unclear. In this study, we identified an ERF‐VII transcription factor, *StRAP2.3*, that directly regulates *StInvInh2* to positively modulate CIS resistance. Acting as a nuclear-localized transcriptional activator, StRAP2.3 directly binds the ACCGAC cis-element in the promoter region of *StInvInh2*, enabling promoter activity. Overexpression of *StRAP2.3* in CIS-sensitive potato tubers induced *StInvInh2* mRNA abundance and increased CIS resistance. In contrast, silencing *StRAP2.3* in CIS-resistant potato tubers repressed the expression of *StInvInh2* and decreased CIS resistance. We conclude that cold-responsive *StInvInh2* is due to the binding of StRAP2.3 to the ACCGAC cis-element in the promoter region of *StInvInh2*. Overall, these findings indicate that StRAP2.3 directly regulates *StInvInh2* to positively modulate CIS resistance, which may provide a strategy to improve the processing quality of potatoes.

## Introduction

Potato chips and French fries are the major value-added processed products of potato tubers. Reducing sugars (RS) in potato tubers are undesirable in these processed products. RS can react with amino acids during frying, resulting in a dark color, bitter taste, and acrylamide accumulation, all of which negatively impact the quality of processed products and food safety^1,2^. To maintain a sustained supply of raw materials, potato tubers are usually stored under cold conditions (<10 °C) to reduce pathogenesis, sprouting, and weight loss. However, a great concern with respect to cold-stored potato tubers is the accumulation of RS, a process known as cold-induced sweetening (CIS). Therefore, the amount of RS in potato tubers must be minimized to guarantee the quality of processed products. The amount of RS that accumulates in cold-stored tubers varies markedly among genotypes and is largely regulated by vacuolar invertase (VI) activity. Variation in VI activity has been identified as the key factor in determining the CIS resistance level of potato tubers^3,4^. Cold-induced *StvacINV1* is the key VI gene involved in regulating the VI activity of cold-stored potato tubers^5–7^. However, *StvacINV1* mRNA abundance is not always associated with RS accumulation in cold-stored tubers^6,8^. Many studies have indicated that VI activity is regulated by inhibitor proteins at the posttranslational level^9–11^. Initially, cDNAs of two invertase inhibitors from tobacco (*Nicotiana tabacum*) were characterized^12,13^. Ectopic expression of one of them, *Nt-inhh*, strongly reduced VI activity and blocked RS accumulation in cold-stored potato tubers^13^. These findings further led to the hypothesis of the occurrence of posttranslational regulation of VI activity by its inhibitor(s) in potato. Emerging evidence has since revealed that *StInvInh2* functions as an inhibitor of *StvacINV1* and plays a pivotal role in regulating CIS in potato tubers by capping VI activity^14,15^. Intriguingly, the *StInvInh2* mRNA abundance in CIS-resistant genotypes was shown to be higher than that in CIS-sensitive genotypes during prolonged cold-storage periods, resulting in varying degrees of reductions in VI activity, leading to varying degrees of CIS^15–17^. This negative relationship between *StInvInh2* mRNA abundance and VI activity seems to be regulated by genotype and to be conditioned by the cold. To explore the possible causes of the various cold-responsive patterns of *StInvInh2* expression in potato genotypes with contrasting CIS ability, small RNAs and their targets were identified in cold-stored tubers via deep sequencing and degradome analysis to test whether post-transcriptional events are involved in this process. However, we did not detect any posttranscriptional events involved in variation in *StInvInh2* mRNA abundance^18^. The promoter of *StInvInh2* was then isolated from various potato genotypes with contrasting CIS abilities, and its activity was analyzed in two of the genotypes. The results showed distinct cold responsiveness of the *StInvInh2* promoter in the two genotypes, suggesting that the active state of transcription factor(s) (TFs) could be one of the causal factors of the diversity of *StInvInh2* gene transcriptional regulation^19^. However, whether and how the expression of *StInvInh2* is regulated by cold-responsive regulators is not clear. Interestingly, the *StInvInh2* promoter contains a dehydration-responsive element/C-repeat (DRE/CRT) motif, which is generally bound by APETALA2/ethylene-responsive factor (AP2/ERF) TFs^19,20^. Therefore, cold-responsive AP2/ERFs could be considered candidate regulators of *StInvInh2* transcription in cold-stored potato tubers. A coexpression model involving two ERFs and *StInvInh2* was constructed based on the comparison of cold-responsive transcription profiles of two potato genotypes with contrasting CIS abilities^21^. One of these *ERFs*, *StRAP2.3*, which is homologous to *RAP2.3* in *Arabidopsis thaliana*, was selected for further study. We found that *StRAP2.3* was coexpressed together with *StInvInh2* in cold-stored tubers and could specifically bind the ACCGAC cis-element of the *StInvInh2* promoter and enable promoter activity. The role of *StRAP2.3* in regulating the CIS of tubers was determined in transgenic potato tubers. Finally, we established that StRAP2.3 directly regulates *StInvInh2* to positively modulate CIS resistance of potato tubers. We propose a regulatory model of the involvement of *StRAP2.3* in the CIS resistance process, increasing our understanding of the transcriptional regulatory mechanism of *StInvInh2* during cold-storage periods.

## Results

### Coexpression of *StRAP2.3* and *StInvInh2* in potato tubers during cold storage

**Fig. 1:**
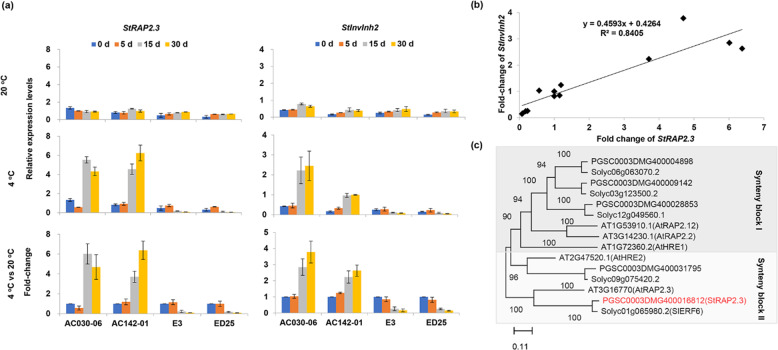
Cold-response patterns of *StRAP2.3* during storage periods and sequence analysis of StRAP2.3. **a** Expression patterns of *StRAP2.3* and *StInvInh2* in potato tubers during 20 °C and 4 °C storage periods, assessed via qRT-PCR. Potato CIS-sensitive genotypes: E3 and ED25; potato CIS-resistant genotypes: AC030-06 and AC142-01. **b** Correlations of fold-changes of *StRAP2.3* and *StInvInh2* in stored tubers. **c** Phylogenetic tree of ERF-VIIs from potato, *Arabidopsis*, and tomato. The tree is based on the alignment of the amino acid sequences using the neighbor-joining method with 1000 bootstraps in Mega X. The expression level of potato ef1α (AB061236) was set as 100 and used for normalization. Each data point represents the mean value of three readings

In our previous transcriptome analysis, expression of the *StRAP2.3* gene, which is a homolog of *A. thaliana RAP2.3*, in tubers exposed to cold conditions exhibited a trend similar to that of *StInvInh2* during storage^21^. The expression patterns of the *StRAP2.3* and *StInvInh2* genes during cold storage were further analyzed via RT-qPCR in potato genotypes with contrasting CIS abilities. The mRNA abundances of both *StRAP2.3* and *StInvInh2* were much higher in the CIS-resistant genotypes than in the CIS-sensitive genotypes during prolonged cold-storage periods. Similarly, the fold-change levels of *StRAP2.3* and *StInvInh2* transcripts were greater in the CIS-resistant genotypes than in the CIS-sensitive genotypes (Fig. 1a). A positive correlation in fold change levels was found between the *StRAP2.3* and *StInvInh2* transcripts (Fig. 1b), implying that *StRAP2.3* may be involved in the regulation of *StInvInh2* expression. The *StRAP2.3* cDNA sequence was then isolated from cold-stored tubers of the CIS-resistant potato genotype AC142-01. *StRAP2.3* has an open reading frame of 795 bp, encoding a 264-aa protein with a predicted protein *M*_r_ of 29.4 kDa (Fig. S1). StRAP2.3 contains a conserved AP2 domain and an N-terminal CMVII domain, which are classic structural features of the ERF‐VII subfamily in all flowering plant species^22,23^. Phylogenetic analysis of ERF‐VII subfamily members in species such as *Arabidopsis*, tomato, and potato clearly classified them into two groups. StRAP2.3 was closely related to AtRAP2.3 and SlERF6, which belong to group II (Fig. 1c).

### StRAP2.3 specifically binds to the ACCGAC cis-element of the *StInvInh2* promoter and enables its activity

**Fig. 2:**
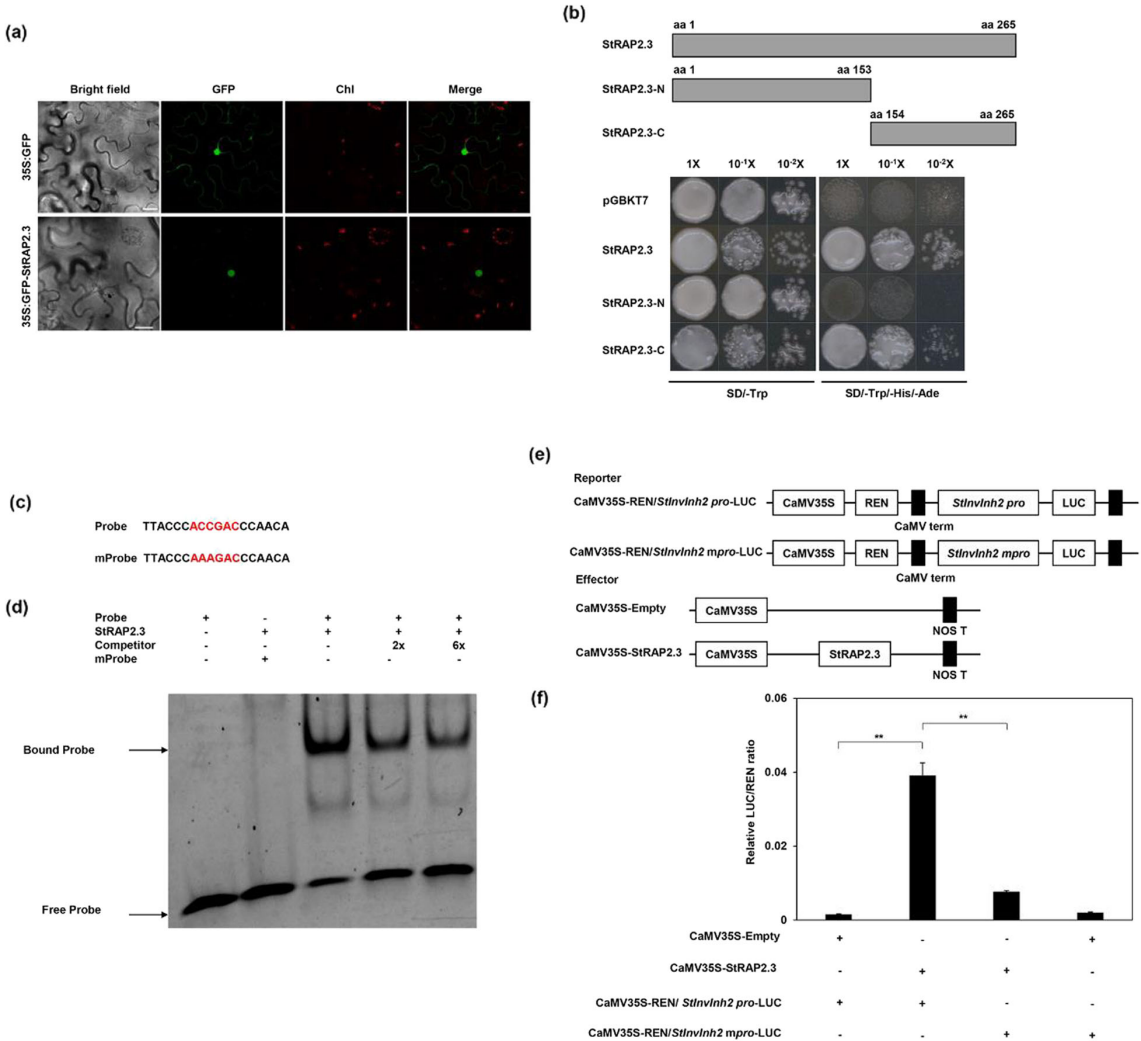
Interaction of StRAP2.3 with the promoter of *StInvInh2*. **a** Subcellular localization of the GFP alone and GFP-StRAP2.3 proteins transiently expressed in tobacco leaves. Bars = 25 μm. **b** Transcriptional activation analysis of StRAP2.3 in yeast. Schematic diagrams of full-length or truncated StRAP2.3 (N-terminal region, StRAP2.3-N; C-terminal region, StRAP2.3-C). Growth of yeast cells (diluted or undiluted) transformed with different constructs on selective media, with pGBKT7 serving as a control. **c** Probes used for the electrophoretic mobility shift assays (EMSAs). The top one is a probe synthesized based on the StInvInh2 promoter sequence, and the bottom one contains AAAGAC instead of ACCGAC. **d** EMSA showing the binding of StRAP2.3 to the StInvInh2 promoter. “+” and “−” indicate the presence and absence, respectively, of the indicated probe or protein; the black arrows indicate the position of proteins and DNA complexes or of free probes. **e** Schematic representation of the reporter and effector plasmids used in the assay. The reporter plasmid contains the genuine promoter or mutant promoter of StInvInh2 fused to LUC luciferase and REN luciferase driven by the CaMV35S promoter. The effector plasmid contains the StRAP2.3 sequence or null sequence driven by the CaMV35S promoter. **f** Dual-luciferase assay showing the relative StRAP2.3 activation of the StInvInh2 promoter. Each value is the mean ± SD of three biological replicates. **represents statistical significance at *P* < 0.01

To determine the subcellular localization of StRAP2.3, a GFP-StRAP2.3 construct was transiently expressed in tobacco (*Nicotiana benthamiana*) leaves. Fluorescence of the GFP-StRAP2.3 protein was detected in the nucleus, while the fluorescence of GFP alone was distributed in both the nucleus and the cytoplasm (Fig. 2a), indicating that StRAP2.3 is localized in the nucleus. To further investigate whether StRAP2.3 has transcriptional activity, a transcription activation assay was performed in yeast. Full-length or truncated fragments of *StRAP2.3* were fused to the GAL4 DNA-binding domain in a pGBKT7 vector (Clontech, Palo Alto, CA, USA). The fusion constructs were then separately transformed into yeast strain AH109. The results showed that all transformants grew well on SD-Trp media. Yeast cells transformed with a pGBKT7 control vector or with a vector containing the N-terminus of StRAP2.3 did not survive on SD/-Trp/-His/-Ade selective media. However, yeast transformants with the full-length or C-terminus of *StRAP2.3* grew vigorously in the same media (Fig. 2b). These results suggest that *StRAP2.3* exhibited transcriptional activity in yeast cells and that the C-terminus of StRAP2.3 is required for this process. Therefore, we confirmed that StRAP2.3 is a TF with intact trans-acting activity.

We sought to test whether the DRE/CRT cis-element could provide a means for StRAP2.3 to regulate *StInvInh2* directly. First, using electrophoresis mobility shift assays (EMSAs), we examined whether StRAP2.3 could bind specifically to the DRE/CRT cis-element of *StInvInh2*. StRAP2.3 proteins fused to His were affinity purified. Two 18-bp oligonucleotides, one containing the genuine cis-element (ACCGAC) and the other containing the mutant cis-element (AAAGAC), were synthesized based on the *StInvInh2* promoter sequence and labeled as the probe and mutant probe, respectively (Fig. 2c). The same oligonucleotide containing the genuine cis-element that was unlabeled was used as a competitor. The results showed that the binding signal was strongly detected when StRAP2.3 was incubated together with the probe and that the signal was decreased markedly when the competitor was added. However, the binding signal was undetectable when StRAP2.3 was incubated together with the mutant probe (Fig. 2d). These results suggest that StRAP2.3 specifically binds to the ACCGAC cis-element in vitro. We then examined whether StRAP2.3 could enable *StInvInh2* promoter activity via a dual-luciferase reporter system in *N. benthamiana* leaves. The dual-luciferase reporter plasmids harbored either the genuine promoter (ACCGAC cis-element) or the mutant promoter (AAAGAC cis-element) of *StInvInh2* fused to LUC or REN driven by the CaMV35S promoter (yielding CaMV35S-REN/*StInvInh2* pro-LUC and CaMV35S-REN/*StInvInh2* mpro-LUC, respectively). The effector plasmid carrying *StRAP2.3*, as well as the empty plasmid, was expressed under the control of the CaMV35S promoter (Fig. 2e). The LUC/REN ratio significantly increased when the *StInvInh2* pro-LUC construct was cotransfected together with the StRAP2.3 effector compared with that of the empty or mutant control (Fig. 2f). Collectively, these results indicate that StRAP2.3, a nuclear-localized transcriptional activator, was able to induce *StInvInh2* expression by specifically binding the ACCGAC element of the *StInvInh2* promoter in *N. benthamiana* leaves.

### StRAP2.3 enhances *StInvInh2* transcription and inhibits VI activity in cold-stored tubers

**Fig. 3:**
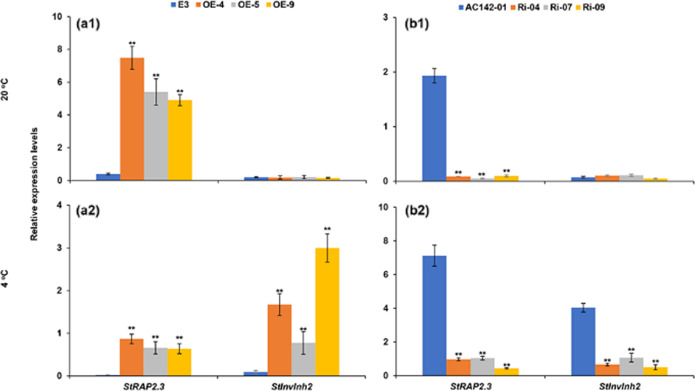
Effects of *StRAP2.3* transcription on *StInvInh2* mRNA abundance in stored potato tubers. **a1**, **b1** Relative expression levels of *StRAP2.3* and *StInvInh2* in tubers of OE and Ri transgenic potato plants at 20 °C storage, respectively. **a2**, **b2** Relative expression levels of *StRAP2.3* and *StInvInh2* in tubers of OE and Ri transgenic potato plants at 4 °C storage, respectively. E3: Potato CIS-sensitive genotype used for overexpression transformation; AC142-01: Potato CIS-resistant genotype used for RNAi transformation. The expression level of potato ef1α (AB061236) was set as 100 and used for normalization. Each data point is the mean value of three readings. The vertical bars represent the standard deviations, **P < 0.01

**Fig. 4:**
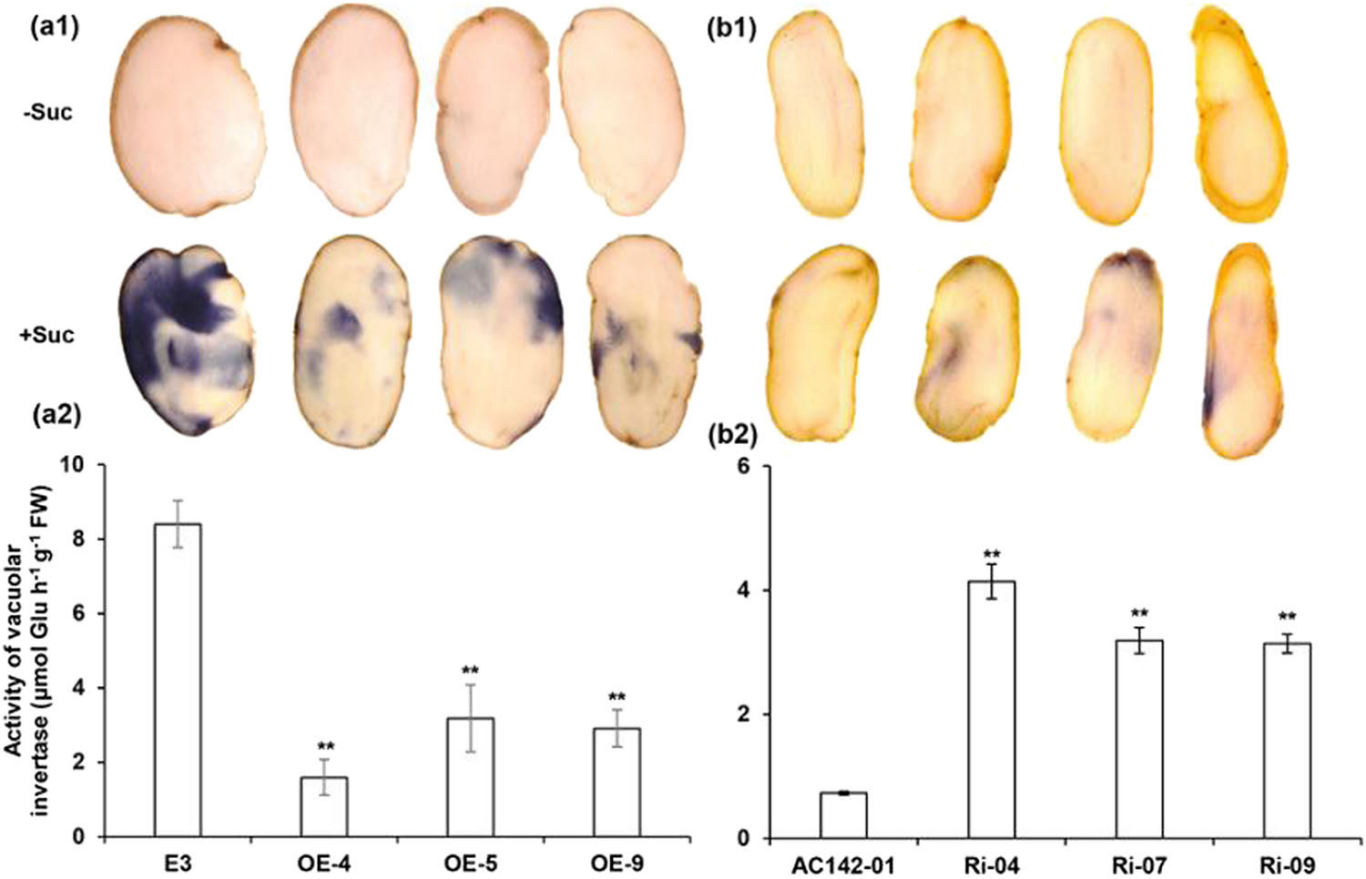
Effects of *StRAP2.3* transcription on VI activity in cold-stored potato tubers of transgenic lines. **a1** NBT histochemical staining indicating reduced acid invertase activity in tubers of OE potato plants. **b1** NBT histochemical staining showing increased acid invertase activity in tubers of Ri potato plants. **a2** Overexpression of *StRAP2.3* significantly decreased the activity of vacuolar invertase. **b2** RNAi of *StRAP2.3* significantly increased the activity of vacuolar invertase. The potato tubers were stored at 4 °C for 30 days. Each data point is the mean value of three readings. The vertical bars represent the standard deviations; **P < 0.01

To determine its physiological role in vivo, *StRAP2.3* was overexpressed via transformation in the CIS-sensitive potato genotype E3 (denoted as the OE line), and *StRAP2.3* was silenced via RNA interference in the CIS-resistant potato genotype AC142-01 (denoted as the Ri line). Plantlets of three OE lines whose transcripts increased by more than 17 times (17.29–26.40) and plantlets of three Ri lines whose transcripts decreased by more than 90% (92.8–99.1%) were selected for detailed characterization (Fig. S2). The transgenic lines showed normal plant morphology and tuber development (similar to those of their corresponding wild-type controls) under greenhouse conditions (Fig. S3). The tubers were harvested and subsequently used for storage experiments. As expected, the expression levels of *StRAP2.3* in the tubers were high in the OE lines (Fig. 3a1, a2) and low in the Ri lines (Fig. 3b1, b2) under both 20 °C and 4 °C storage conditions. However, *StInvInh2* expression was strongly affected under only 4 °C conditions in the tubers of both the overexpression and silenced transgenic plants (Fig. 3a2, b2). These results indicate that *StRAP2.3* is involved in cold-dependent *StInvInh2* regulation in tubers. Since StInvInh2 can specifically inhibit the activity of StvacINV1^15^, StvacINV1 activities were further analyzed in cold-stored tubers of both the OE and Ri transgenic plants. First, changes in acid invertase activity were evaluated via histochemical activity staining in situ. Nitro blue tetrazolium (NBT) staining of the OE lines resulted in a weak blue color visible in cold-stored tubers, suggesting suppressed acid invertase activity (Fig. 4a1). Conversely, NBT staining of the Ri lines resulted in a strong blue color visible in cold-stored tubers, suggesting elevated acid invertase activity (Fig. 4b1). Acid invertase activity was then assayed using an in vitro enzyme assay. The results showed that VI activity decreased by 62.1–81.1% in the OE lines and increased by 4.30–5.67 times in the Ri lines (Fig. 4a2, b2). Taken together, these results indicate that StRAP2.3 increased *StInvInh2* transcription to inhibit VI activity.

### StRAP2.3 positively regulates CIS resistance and processing quality

Sugar content analysis revealed that changes in the RS and sucrose contents in the transgenic lines were not obviously different during storage at 20 °C (Table 1). The RS contents were lower in the OE tubers and higher in the Ri tubers when compared to those of their relative wild type during 4 °C storage (Table 1), which is in accordance with the levels of VI activity shown in Fig. 4. A dramatically higher sucrose/RS ratio was observed in the OE tubers, whereas this ratio was notably lower in the Ri tubers when compared to the ratio of their relative wild type (Table 1), demonstrating that *StRAP2.3* is primarily associated with VI-catalyzed sucrose degradation in cold-stored tubers. The expression of other key genes involved in the starch-sugar interconversion pathway was also analyzed via RT-qPCR; no obvious difference was detected in the transcript abundances of *StvacINV1*, *AGPase*, *BMY*s, *AMY*, *SPS*, and so on (Fig. S4), indicating that StRAP2.3 does not influence the expression of these genes in potato tubers.

**Table 1:** Sugar contents of transgenic and wild-type tubers stored at 4 °C and 20 °C for 30 days

Line	20 °C			4 °C		
	RS content (mg g^−1^ FW)	Sucrose content (mg g^−1^ FW)	Sucrose/RS	RS content (mg g^−1^ FW)	Sucrose content (mg g^−1^ FW)	Sucrose/RS
E3	0.53 ± 0.01	1.65 ± 0.05	3.09	6.05 ± 0.46	1.40 ± 0.18	0.23
OE-4	0.48 ± 0.05	1.39 ± 0.28	2.87	1.34 ± 0.08^**^	4.20 ± 1.2^**^	3.13^**^
OE-5	0.68 ± 0.01	1.94 ± 0.27	2.84	1.22 ± 0.26^**^	6.60 ± 0.8^**^	5.41^**^
OE-9	0.61 ± 0.10	1.74 ± 0.14	2.86	1.52 ± 0.08^**^	2.82 ± 0.1^**^	1.86^**^
AC142-01	0.22 ± 0.03	0.25 ± 0.03	1.14	0.78 ± 0.05	3.50 ± 0.31	4.49
Ri-04	0.17 ± 0.02	0.23 ± 0.09	1.37	2.03 ± 0.12^**^	2.31 ± 0.23^**^	1.34^**^
Ri-07	0.20 ± 0.04	0.21 ± 0.07	1.05	1.70 ± 0.05^**^	1.89 ± 0.13^**^	1.11^**^
Ri-09	0.22 ± 0.01	0.23 ± 0.01	1.04	2.63 ± 0.18^**^	2.47 ± 0.25^**^	0.94^**^

E3: CIS-sensitive potato genotype used for overexpression transformation; AC142-01: CIS-resistant potato cultivar used for RNAi transformation. The data are the means ± SDs of three biological replicates. **represents a statistical significance at *P* < 0.01

**Fig. 5:**
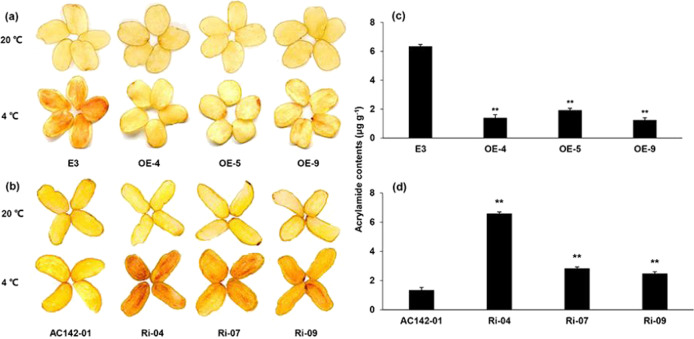
Color of and acrylamide content in chips from transgenic and nontransgenic potato tubers. **a**, **b** Color of chips from OE and wild-type E3 tubers, as well as Ri and wild-type AC142-01 tubers stored separately at 4 °C and 20 °C for 30 days. **c**, **d** Acrylamide content of potato chips processed from tubers stored at 4 °C for 30 days. Each value is the mean ± SD of three biological replicates. **represent a statistical significance at *P* < 0.01

The tubers were then subjected to frying to evaluate the effects of *StRAP2.3* on chip quality. Few variations in chip color were observed between the transgenic tubers and their corresponding wild-type controls, which had been stored at 20 °C for 30 days. However, the OE tubers displayed a much lighter chip color than the wild-type control E3 tubers did when the tubers were stored at 4 °C for 30 days (Fig. 5a), whereas the Ri tubers showed an obviously darker chip color than did the wild-type control AC142-01 tubers (Fig. 5b). In addition, the acrylamide content in the chips from the OE tubers was reduced by 69.6–80.4% compared with that from the wild-type E3 tubers (Fig. 5c), while it increased by 1.86–4.98 times in the chips of the Ri tubers compared with the wild-type AC142-01 tubers (Fig. 5d). These results indicate that *StRAP2.3* is an important player in the process of CIS resistance in potato tubers.

## Discussion

RS accumulation in cold-stored potato tubers negatively affects their processing quality. Therefore, understanding the mechanisms that regulate CIS resistance is of great significance to the potato processing industry. StInvInh2 is known to play a crucial role in CIS resistance by inhibiting VI activity^15,18^. The diversity of cold-response patterns of *StInvInh2* mRNA abundance in potato genotypes with contrasting CIS abilities could not have resulted from either posttranscriptional events or different sequences of the *StInvInh2* promoter^18,19^. We propose that the active state of TFs binding to the promoter of *StInvInh2* could be a causal factor. To identify candidate TFs regulating *StInvInh2*, the *StInvInh2* promoter sequence was used as bait to screen a cold-stored potato tuber cDNA library by yeast one-hybrid assays. Unfortunately, the experiment failed because of the strong autoactivation activity of the bait in yeast cells. Alternatively, cold-responsive ERF TFs were considered candidate regulators of *StInvInh2* expression in cold-stored potato tubers because the *StInvInh2* promoter contains a DRE/CRT motif that can bind ERF TFs. A cold-responsive CIP353 encoding an ERF-domain protein, which is a homolog of *A. thaliana* RAP2.3, has been isolated from cold-stored potato^24^. Similar expression patterns between this gene and *StInvInh2* were also identified based on a comparison of the expression profiles of potato genotypes with contrasting CIS ability during cold storage^21^. Therefore, it was reconsidered to be a candidate protein and named StRAP2.3 because it regulates *StInvInh2* expression.

RT-qPCR verified that *StRAP2.3* expression was induced by prolonged cold in CIS-resistant genotypes (Fig. 1a), which is consistent with the findings in a previous study^24^. The abundance of *StRAP2.3* was higher in CIS-resistant genotypes than in CIS-sensitive genotypes in cold-stored tubers (Fig. 1a). The *StRAP2.3* expression patterns are consistent with those of *StInvInh2* in both potato genotypes, in contrast to CIS ability^11^ (Fig. 1a, b), suggesting that StRAP2.3 may be a candidate TF regulating *StInvInh2* expression and RS accumulation in cold-stored tubers. We subsequently obtained direct evidence to support this idea. First, StRAP2.3 directly binds to the promoter of *StInvInh2* and activates it, as verified via EMSA and dual-luciferase assay approaches (Fig. 2d, f). Furthermore, overexpression of *StRAP2.3* in cold-stored potatoes resulted in an increase in *StInvInh2 mRNA* abundance. In contrast, *StRAP2.3* in the Ri potato tubers (Fig. 3b2) provided additional evidence that *StRAP2.3* activated *StInvInh2* expression. Therefore, it is reasonable to indicate that StRAP2.3 positively regulates *StInvInh2* expression in cold-stored tubers. Finally, the index of VI activity, sugar accumulation, chip color, and acrylamide content associated with CIS were evaluated in cold-stored tubers of the transgenic lines. Compared with the E3 wild-type tubers, the OE tubers presented lower VI activity and RS accumulation, lighter chip color, and lower acrylamide content. In contrast, the tubers of the Ri lines have higher VI activity and RS accumulation, darker chip color, and higher acrylamide content than the wild-type AC142-01 tubers do. These results are consistent with those of our previous study about the role of *StInvInh2* in CIS resistance^15^. Collectively, StRAP2.3 positively regulates CIS resistance by activating *StInvInh2*.

Group VII ERFs are plant-specific TFs that have been determined to be important regulators of biotic and abiotic stress responses^25^. They have previously been shown to bind to a range of promoter DNA motifs, including GCC-boxes^26^, 5ʹ-ATCTA-3ʹ sequences^27^, and hypoxia-responsive promoter elements^28^, suggesting that they have various gene targets. In *Arabidopsis*, AtRAP2.3 interacts with the GCC-box of the *ABI5* promoter and promotes *ABI5* expression in the mature seed endosperm to maintain seed dormancy^26^, and it positively regulates sugar metabolism and the expression of hormone signal-related genes to improve tolerance to different stresses^29^. *SlERF6* negatively regulates carotenoid accumulation only in fruits and not in leaves, suggesting that this gene may function under tissue-specific constraints^30^. In this study, EMSAs and dual-LUC assays verified the StRAP2.3 interaction together with the DRE/CRT motif (Fig. 2) and showed that enhanced ERF-VIIs have various gene targets. StRAP2.3 positively regulates *StInvInh2* expression in cold-stored tubers but not in room-temperature (20 °C)-stored tubers (Fig. 3), suggesting that the function of this gene may also be under cold-specific constraints. One possible explanation might involve a distinct chromatin environment with greater accessibility, which may facilitate access to StRAP2.3, which is required for *StInvInh2* regulation in response to cold^31^.

## Conclusions

**Fig. 6:**
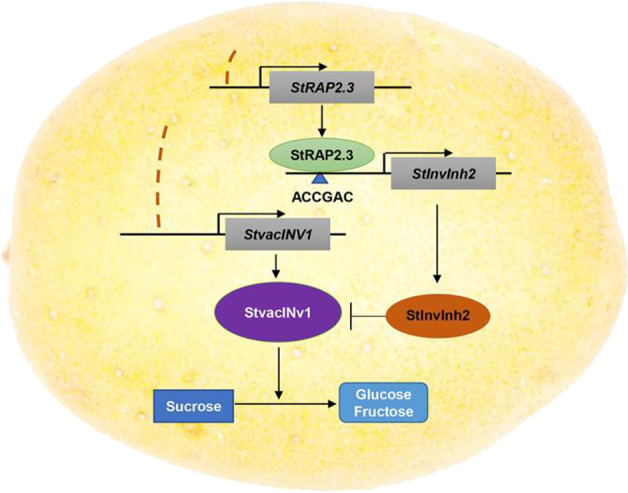
Proposed model of the mechanism through which CIS resistance is regulated through *StRAP2.3* in potato. *StRAP2.3* transcription was induced in CIS-resistant genotypes by prolonged cold storage, and StRAP2.3 directly bound the ACCGAC cis-element in the promoter region of *StInvInh2* to activate *StInvInh2* expression. StInvInh2 binds to StvacINV1 to form an inactive complex that reduces sucrose degradation^15^

In this study, we identified a potato ERF-VII subfamily member named *StRAP2.3*, which directly activates the expression of the *StInvInh2* gene to enhance CIS resistance (Fig. 6). The manipulation of *StRAP2.3* expression may therefore be useful for regulating CIS resistance. Thus, this study describes a new ERF regulon that enables us to understand the physiological relevance of ERF proteins in CIS resistance. In the future, we will try to analyze sequence polymorphisms and the mRNA abundance of *StRAP2.3* among various potato genotypes with contrasting CIS abilities to learn more about the role of this gene in the regulation of CIS resistance in potato tubers. Our results indicate that StRAP2.3 directly regulates *StInvInh2* to positively modulate CIS resistance, which may provide an avenue to improve the processing quality of potatoes.

## Experimental procedures

### Plant materials and treatment

The plantlets of CIS-resistant clones (AC142-01 and AC030-06), CIS-sensitive clones (ED25 and E3), and transformed potato lines were maintained vegetatively in vitro, propagated through single-node cuttings on semisolid (7 g L^−1^ agar) MS basal media supplemented with 4% sucrose and incubated at 20 ± 1 °C under a 16/8 h day/night photoperiod (light intensity of 83 μmol m^−2^ s^−1^) at the Chongqing Key Laboratory of Biology and Genetic Breeding for Tuber and Root Crops. Four-week-old plantlets were grown in Ø 24 cm pots in a greenhouse at 18–25 °C and supplemented with 300 μmol m^−2^ s^−1^ of light under a 12 h photoperiod at Southwest University in Chongqing in 2015 and 2016. The resulting mature tubers were treated as previously described^15^.

### Gene isolation and sequence analysis

The coding region without the stop code of *StRAP2.3* was amplified with specific primers (Table S1) and cloned into a pENTR/D cloning vector (Invitrogen, Carlsbad, CA). Sets of related ERF-VII amino acid sequences from potato^32^, tomato^23^, and *Arabidopsis*^23^ were downloaded from genome databases. Multiple alignments were performed using the ClustalX program and displayed by Jalview. A phylogenetic tree was constructed using MEGA X software with the neighbor-joining method. The reliability of the tree was ensured via 1000 bootstrap replicates.

### Subcellular localization analysis

The full-length coding sequence (without the termination codon) of *StRAP2.3* was amplified with gene-specific primers (Table S1) and subcloned into a PH7LIC-N-eGFP plasmid, yielding a GFP-StRAP2.3 construct. The construct was subsequently transformed into *Agrobacterium tumefaciens* GV3101. Positive colonies were transiently expressed in tobacco (*Nicotiana benthamiana*) by agroinfiltration as previously described^33^.

### Transactivation activity assays in yeast

Full-length or truncated (N, amino acids 1–124; C, amino acids 125–248) *StRAP2.3* CDSs were amplified with specific primers (Table S1) and subcloned into pGBKT7 vectors (Clontech, Palo Alto, CA, USA) to produce BD plasmids. The recombinant vectors were introduced into AH109 yeast cells by the lithium acetate-mediated method. Positive transformants with different dilutions were then spotted onto SD/-Trp and SD/-Trp-His-Ade media. pGBKT7 was used as a negative control.

### Electrophoretic mobility shift assays

*StRAP2.3* was subcloned into a pET32a vector to construct a plasmid for the expression of recombinant His-StRAP2.3 protein in Rosetta *E. coli* (Novagen, Madison, WI, USA). The protein was expressed in Rosetta *E. coli* by induction with 0.2 mM isopropyl-β-D-thiogalactoside (IPTG) for 16 h at 16 °C. His-tagged proteins were purified using Ni-NTA magnetic agarose (Qiagen, Valencia, CA) according to the manufacturer’s instructions. An 18-bp oligonucleotide containing the ACCGAC element was synthesized and labeled with FAM luciferase; in addition, the same or mutated fragments were used as competitors. Purified His-tagged proteins were incubated with the FAM-labeled probes and competitors for 15 min on ice. The resulting reaction mixtures were then subjected to a native 40% (w/v) polyacrylamide gel, and electrophoresis was performed at 4 °C using 1× Tris-glycine buffer for 1 h at 100 V. The gels were imaged with an Amersham Imager 600 (GE Healthcare, Pittsburgh, PA, USA).

### Dual-luciferase reporter assays

The promoter and mutant promoter sequences (those with a ACCGAC cis-element and AAAGAC cis-element, respectively) of *StInvInh2* were amplified and subcloned into a pGreenII 0800-LUC reporter vector. A 35S:*StRAP2.3* construct was used as an effector, and an empty vector was used as the negative control. The reporter and effector constructs were transiently expressed in tobacco (*Nicotiana benthamiana*) leaves by agroinfiltration. LUC and REN activities were detected using a dual-luciferase reporter assay system (E710, Promega, Madison, WI, USA). At least three biological replicates were used for each combination. The activity of the promoters was expressed as the ratio of LUC to REN.

### Vector construction and plant transformation

The full-length or RNAi fragments of *StRAP2.3* CDS were amplified with specific primers (Table S1). The full-length CDS and RNAi fragments were then subcloned into a pJCV55 overexpression vector and a pHELLSGATE8 vector using the recombination method^34^, respectively. The overexpression and RNAi constructs were subsequently transformed into potato genotypes E3 and AC142-01, respectively, via *Agrobacterium*-mediated transformation, as previously described^15,35^.

### RNA isolation and quantitative RT-PCR

Total RNA was extracted from tubers and leaves using an RNAprep Pure Plant Plus Kit (Polysaccharides & Polyphenol-rich) (Tiangen, Beijing, China). The RNA was subsequently reverse transcribed into cDNA using Hifair 1st Strand cDNA Synthesis SuperMix (gDNA digester plus) (Yeasen, Shanghai, China). Quantitative real-time PCR (qRT-PCR) was performed using ChamQ^TM^ Universal SYBR qPCR Master Mix (Vazyme, Nanjing, China), and real-time qRT-PCR was performed on a BIO-RAD CFX Connect Real-Time System (Bio-Rad, Hercules, USA). The potato gene *ef1α* (GenBank accession No. AB061263) was selected as an internal reference gene for normalization^36^.

### Invertase activity, sugar content, chip color, and acrylamide content analyses

Frozen tubers were ground into a fine powder in liquid nitrogen for analysis of the activities of vacuolar invertase and sugar content^11^. Staining for invertase activity was performed as described by Su et al.^37^, and chip color and acrylamide analyses were performed as previously described^6^.

### Statistical analysis

Three experiments were performed for each sample, and the data are presented as the means ± SDs. The significance between the treatments was tested by ANOVA using SPSS 13.0 software for Windows (SPSS, Inc., Chicago).

## Supplementary information

Supplementary Materials R1
