# Supplementary material for: StRAP2.3, an ERF‐VII transcription factor, directly activates StInvInh2 to enhance cold-induced sweetening resistance in potato
Source: Hortic Res. 2021 Apr 1;8:82. doi: 10.1038/s41438-021-00522-1 (PMC8012585; doi:10.1038/s41438-021-00522-1)
Supplement: Supplementary file 1 — Supplementary Materials R1 [file 41438_2021_522_MOESM1_ESM.docx]

**Table S1 Primers used in the present study**

| Gene name | Gene ID | Primer F（5’-3’） | Primer R（5’-3’） | Application |
| --- | --- | --- | --- | --- |
| *StRAP2.3* | PGSC0003DMG400016812 | CACCATGTGTGGAGGTGCCATAATCT | ATAGAACTGATGCTGAGTTGAAGCA | TOPO Clone |
| *StRAP2.3* | PGSC0003DMG400016812 | ATTACGCCGAGGTCATGTGTGG  AGGTGCCATAATC | TAGGGAAGAGGTCAATAGAACT  GATGCTGAGT | Subcellular localization |
| *StRAP2.3* | PGSC0003DMG400016812 | GGCCATGGAGGCCGAATTCA  TGTGTGGAGGTGCCATAAT | GCGGCCGCTGCAGGTCGACT  CAATAGAACTGATGCTGAG | Transactivation activity |
| *StRAP2.3* | PGSC0003DMG400016812 | GGCCATGGAGGCCGAATTC ATGTGTGGAGGTGCCATAAT | GCGGCCGCTGCAGGTCGAC GGGGAAGTTGAGTTTGGCCT | Transactivation activity |
| *StRAP2.3* | PGSC0003DMG400016812 | GGCCATGGAGGCCGAATTC GTCCCATCACCACCTGCTAA | GCGGCCGCTGCAGGTCGAC TCAATAGAACTGATGCTGAG | Transactivation activity |
| *StRAP2.3* | PGSC0003DMG400016812 | CTGATATCGGATCCGAATTC AAGTACAGAGGAATAAGGCA | TGGTGGTGGTGGTGCTCGAG GGGGAAGTTGAGTTTGGCCT | Protein expression |
| *StRAP2.3* | PGSC0003DMG400016812 | CACCAGTAGTGGACCAAGGCTGAGAAAG | GGGGAAGTTGAGTTTGGCCT | TOPO Clone |
| *StInvInh2 pro* | PGSC0003DMG400004616 | ATAGGGCGAATTGGGTACCCCCTTT  ATACTAATGGACAT | *TTTTTGGCGTCTTCCATGG*TG  AGAATTGAAGAAATGAGA | Reporter construction |
| *StInvInh2 mpro* | PGSC0003DMG400004616 | ATAGGGCGAATTGGGTACCCCCTTT  ATACTAATGGACAT | TATCCGTCGATCTGATATTTAA  TAAATGTTGGGTCTTTG | Reporter construction |
| *StInvInh2 mpro* | PGSC0003DMG400004616 | CAAAGACCCAACATTTATTAAATATCA  GATCGACGGATA | TTTTTGGCGTCTTCCATGGTGAG  AATTGAAGAAATGAGA | Reporter construction |
| *StvacINV1* | PGSC0003DMG400013856 | GAATGGAGCAGCACGACTCTT | GCCGACTCAAGTGACCAAATC | RT-qPCR |
| *StInvInh2* | PGSC0003DMG400004616 | CCCTACAATCCGATCCACGTA | TTCACCACGTCCACCATGAT | RT-qPCR |
| *StRAP2.3* | PGSC0003DMG400016812 | GATCCTCAGAAGGGTGTCCG | TGTCACCACGAATGCGCTTA | RT-qPCR |
| *StAGPase* | PGSC0003DMG400000735 | CCCCTGAATCGTCACATTGC | CTTCCCCGGGTGTCTGAGT | RT-qPCR |
| *StGBSS* | PGSC0003DMG400012111 | TGTCATGGACGCAAAACCTTT | AGTCTGCCGATGAAGCCAAT | RT-qPCR |
| *StSSS3* | PGSC0003DMG400016481 | CTTGGTACGATGGTCGGGAT | TTGTTCCATGACCTGCTTGC | RT-qPCR |
| *StBAM1* | PGSC0003DMG400001549 | TGAGATGCGTGACCATGAGC | CAAGTGGAACTTGCGCTTCC | RT-qPCR |
| *StBAM9* | PGSC0003DMG400010664 | CCTTGATGGAAAGACTCCGG | AGAAACGCCCGTGATTGTG | RT-qPCR |
| *StAMY23* | PGSC0003DMG400009891 | GGCATACACAGCCGTTCATCT | ATCCGTCCCCAATCTTCACG | RT-qPCR |
| *EF1α* | AB061263* | ATTGGAAACGGATATGCTCCA | TCCTTACCTGAACGCCTGTCA | RT-qPCR |

*Gene from the National Center of Biotechnology Information database

**Supplementary Figure and Figure legends**

**Figure S1**

**
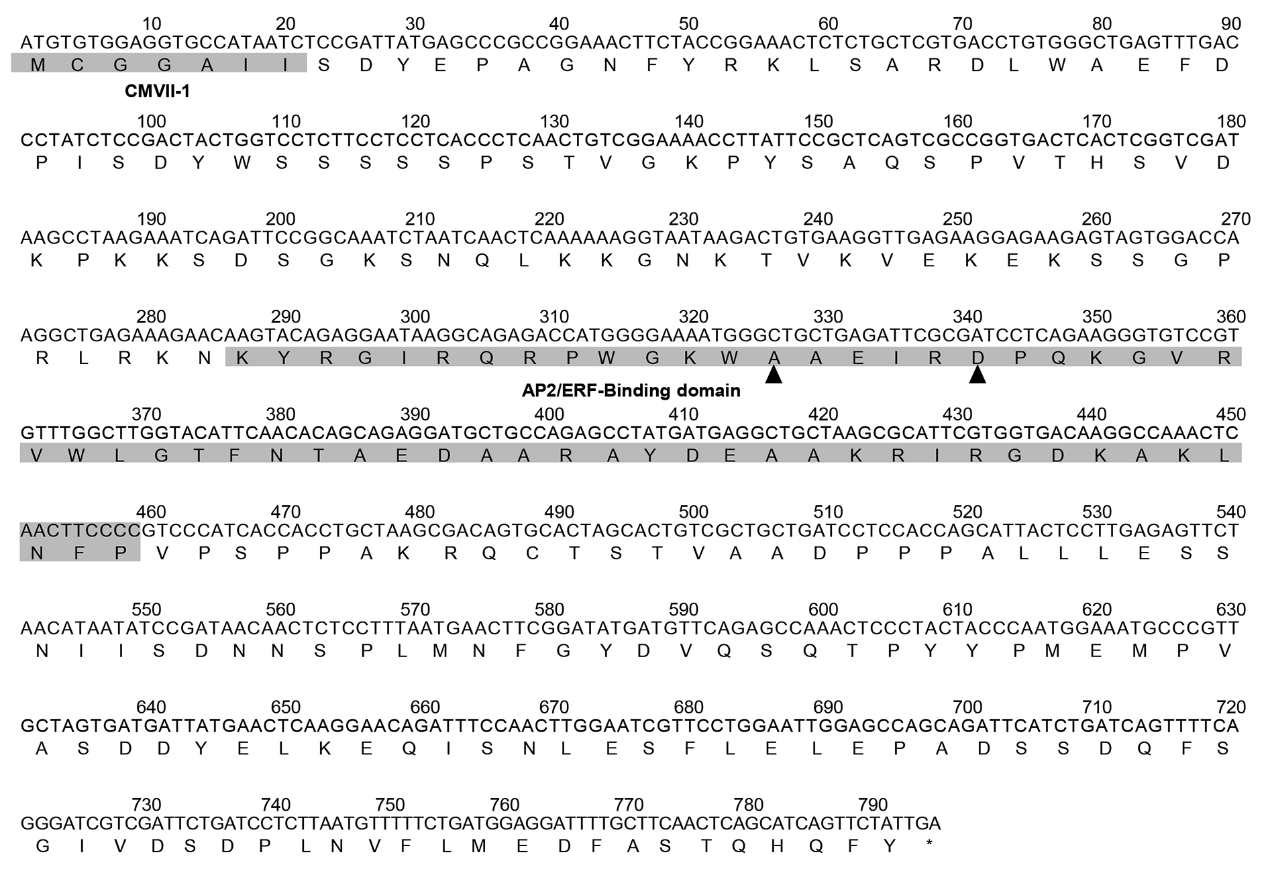
**

**Fig. S1 Sequence analysis of StRAP2.3 in potato**

ORF of *StRAP2.3* and deduced amino acid sequence. Two conserved domains, CMVII-1 of 7 aa and AP2/ERF binding domain of 58 aa were indicated by arrow lines. ▼: Conserved amino acid site.

**Figure S2**

**
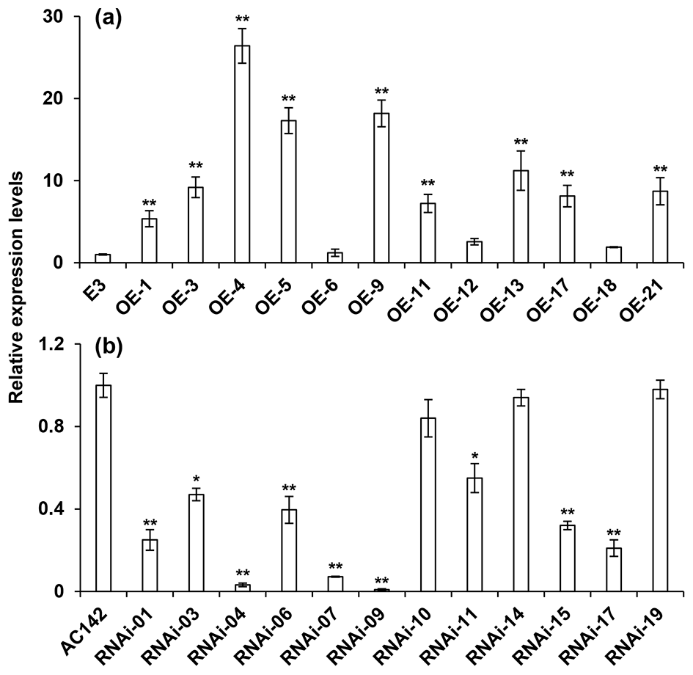
**

**Fig. S2 The expression level of *StRAP2.3*** **in transgenic plantlets**

(a) Transcripts of *StRAP2.3* in OE lines. (b) Transcripts of *StRAP2.3* in Rilines. E3: Potato CIS-sensitive cultivar E3 used for overexpression transformation; AC142-01: Potato CIS-resistant cultivar used for RNAi transformation. The expression in wild-type E3 and AC142-01 was taken as 1 for calculating the fold changes in *StRAP2.3* expression in corresponding transgenic lines. Data were the mean ±SD of three biological replicates. * and ** represent statistical significance at p < 0.05 and p < 0.01, respectively.

**Figure S3**

**
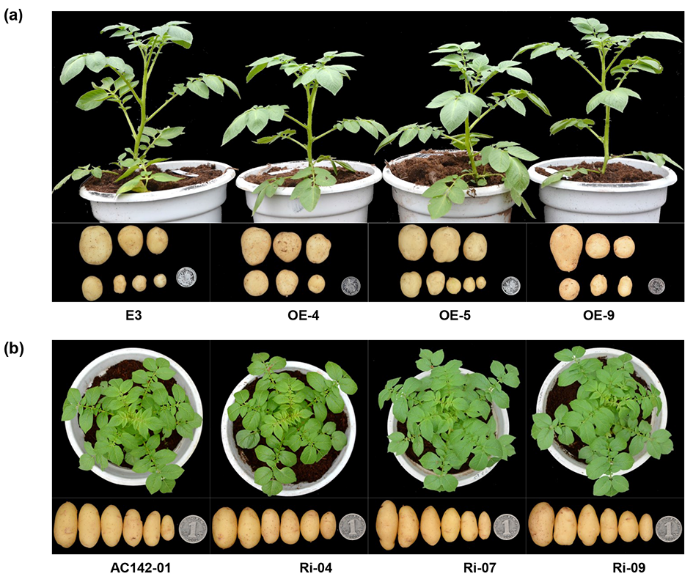
**

**Fig. S3 Phenotypes of representative transgenic lines and controls**

(a) Phenotypes of representative OE lines and E3 controls. (b) Phenotypes of representative RNAi lines and Ac142-01 controls.

**Figure S4**

**
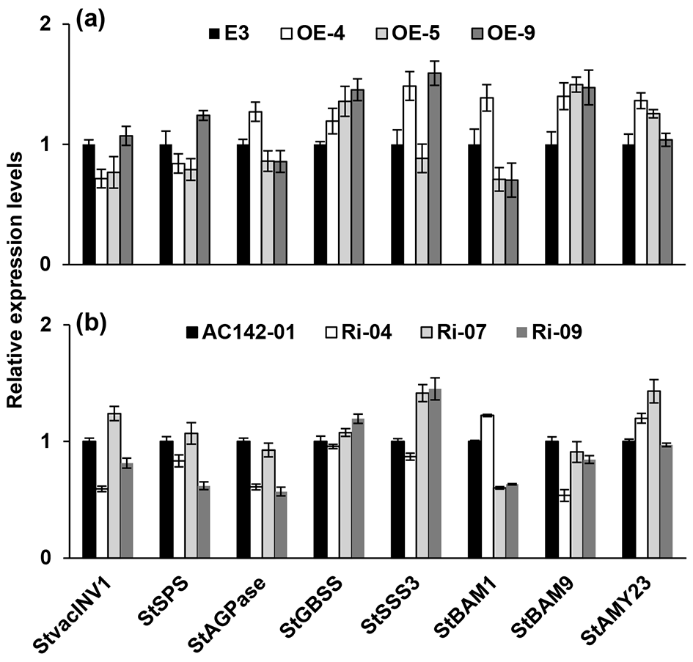
**

**Fig. S4 The expression level of 8 genes involved in starch-sugar intervention pathways of potato tubers during 4°C storage assessed by qRT-PCR.**

Relative expression levels of 8 genes in OE (a) and Ri (b) potato tubers during storage 4 °C storage were analyzed by qRT-PCR, respectively. *StvacINV1*: vacuolar invertase; *StSPS*: sucrose-phosphate synthase isoform B; *StAGPase*: ADP-glucose pyrophosphorylase; *StGBSS*: granule-bound starch synthase; *StSSS3*: soluble starch synthase 3; *StBMY*: beta-amylase; *StAMY*: alpha-amylase. The expression in wild-type E3 and AC142-01 was taken as 1 for calculating the fold changes in 8 genes expression in corresponding transgenic lines. Data were the mean ±SD of three biological replicates.
